# Evidence for the low recording of weight status and lifestyle risk factors in the Danish National Registry of Patients, 1999–2012

**DOI:** 10.1186/s12889-015-2670-9

**Published:** 2015-12-30

**Authors:** Mette Søgaard, Uffe Heide-Jørgensen, Mette Nørgaard, Søren P. Johnsen, Reimar W. Thomsen

**Affiliations:** Department of Clinical Epidemiology, Aarhus University Hospital, Olof Palmes Allé 43-45, 8200 Aarhus, Denmark

**Keywords:** Lifestyle, Overweight, Smoking, Physical inactivity, Alcohol, Registration, Completeness

## Abstract

**Background:**

To examine the prevalence of lifestyle diagnosis codes recorded in the Danish National Registry of Patients (DNRP).

**Methods:**

We identified all hospital contacts in Denmark 1999–2012 with a diagnosis of overweight, obesity, physical inactivity, current tobacco smoking, and/or excessive alcohol consumption. We computed the annual prevalence per 1000 hospital contacts of these diagnoses overall and by baseline characteristics.

**Results:**

Among 56,665,048 hospital contacts, the overall prevalence of recording per 1000 hospital contacts was 4.87 for a diagnosis of obesity, 2.36 for overweight, 2.90 for smoking, 0.39 for excessive alcohol consumption, and 0.47 for physical inactivity. Between 1999 and 2012, marked increases were noted for the prevalence of recorded obesity (30-fold, from 0.26 to 8.02), smoking (26-fold, from 0.18 to 4.88), and overweight (14-fold, from 0.23 to 3.52). Diagnosis coding of excessive alcohol consumption and physical inactivity remained at a very low level. The prevalence of recorded lifestyle risk factors varied substantially according to geographical regions, type of hospital contact, patient age, sex and underlying disease. In 2012, the prevalence of codes for obesity were highest among patients with diabetes (15.64 per 1000), COPD (12.95 per 1000), and congestive heart failure (11.24 per 1000). Codes for smoking were prevalent among patients with COPD (14.11 per 1000), liver disease (12.68 per 1000), and peripheral vascular disease (8.52 per 1000).

**Conclusion:**

Despite increasing prevalence of adverse lifestyle risk factors recorded in the DNRP, the much higher prevalence of similar lifestyle risk factors in health surveys suggests that the completeness of coding in the DNRP remains poor.

**Electronic supplementary material:**

The online version of this article (doi:10.1186/s12889-015-2670-9) contains supplementary material, which is available to authorized users.

## Background

During the last decades, the global disease burden has shifted from communicable to noncommunicable diseases including cardiovascular diseases, diabetes, cancers, and chronic respiratory diseases [1]. Worldwide, noncommunicable diseases were responsible for 54 % of all disability-adjusted life years [2] and 65 % of all deaths in 2010 [3]. Smoking, excessive alcohol use, unhealthy diet, and physical inactivity/obesity are the “big four” modifiable risk factors of this epidemic of noncommunicable diseases [4]. Therefore, strategies for prevention include lifestyle modification and adoption of healthy behaviors. In this context, physicians can play an important role through raising awareness and providing advice to patients with unhealthy behaviors. Nonetheless, prior studies suggest that physicians generally counsel only a minority of patients [5–8].

According to Hospital Accreditation Standards in Denmark and elsewhere, all inpatients and outpatients should be screened with regard to unhealthy lifestyle factors and offered intervention if their adverse lifestyle may influence treatment outcome or otherwise pose a risk for the patient [9]. Yet, it is unclear whether such screening actually occurs at hospitals and to which extent the results are recorded in hospital patient registries over time. Documentation of life style risk factors is important in order to provide continuity in care and to facilitate awareness of these factors during transitions of patient care. Therefore, we examined the prevalence of hospital contacts with a recorded diagnosis code for overweight, obesity, physical inactivity, tobacco, or alcohol consumption in the Danish National Registry of Patients (DNRP) from 1999 through 2012 and assessed whether this registration varied across Denmark’s geographical regions, by type of hospital contact, and according to patient age, sex, and underlying disease.

## Methods

### Setting and study population

Denmark has 5.6 million inhabitants, and the National Health Service provides universal tax-supported health care for all residents, including free access to primary care and hospitals. The country is divided into five regions which have the main responsibility for the provision of public hospital services – both somatic and psychiatric hospitals. Since 1977 the DNRP has tracked each hospital admission in Denmark and recorded dates of admission and discharge and up to 20 discharge diagnoses. The registry covers 99.4 % of all discharge records from Danish hospitals [10]. It allows for one principal diagnosis code given to the condition that prompted the patient’s admission and the main condition responsible for the completed diagnosis and treatment course and up to 20 secondary codes. The secondary diagnoses are given to conditions that coexist at the time of hospital admission or that develop during the hospital stay with no information as to which disease occurred first. The diagnoses are coded by physicians using the Danish version of the International Classification of Diseases, 8th revision (ICD-8) (1977–1993) and 10th revision (1994 onward). Since 1995, visits at hospital outpatient clinics and emergency rooms have been recorded in addition to the inpatient hospital stays.

### Assessment of lifestyle risk factors

Data for this study were obtained for the period from 1999 through 2012. We identified all inpatient and outpatient hospital contacts with a recorded ICD-10 code for overweight, obesity, smoking, excessive alcohol consumption, and physical inactivity in the DNRP. Information on the patient’s lifestyle risk factors are usually obtained by physicians or nurses through patient interview and examination at the time of hospital admission. In 2005, the Danish National Board of Health initiated a project aiming to strengthen the prevention of lifestyle-related diseases through systematic registration of lifestyle risk factors [11]. In relation to this project, additional codes for recording of lifestyle risk factors were implemented along with a list of clinical questions and definitions for the individual risk factors in order to guide physicians and nurses when obtaining this information.

### Statistical analysis

We computed the prevalence of all contacts (e.g., patients could be included in both the numerator and denominator more than once) with one or more of these codes recorded either as primary or secondary diagnoses per 1000 hospital contacts. We computed the prevalence overall and according to study year, health care region, contact type (inpatient vs. outpatient), age, gender, and underlying disease defined as the principal discharge diagnosis recorded in the DNRP. We assessed the recording of lifestyle risk factors in relation to the following underlying diseases: Myocardial infarction, congestive heart failure, peripheral vascular disease, cerebrovascular disease, chronic obstructive pulmonary disease (COPD), diabetes, cancer, and liver disease. These diseases were chosen because they are strongly associated with the examined lifestyle risk factors. We would therefore expect one or more of the lifestyle risk factors to be prevalent in patients with these underlying diseases. To examine to which extent lifestyle risk factors were coded at first hospital contacts, we further restricted the analyses to patients with no hospital contacts within 10 years preceding the date of index admission. The ICD-10 codes used in the study appear in Additional file 1: Table S1. Analyses were performed using Statistical Analysis Software (v 9.2; SAS Institute, Inc, Cary, NC, USA).

## Results

From 1999 through 2012, 56,665,048 hospital contacts occurred among 6,664,495 individual patients in Denmark, of which 39,244,611 (69 %) were outpatient clinic contacts and 17,420,437 (31 %) were inpatient hospitalization contacts.

**Fig. 1:**
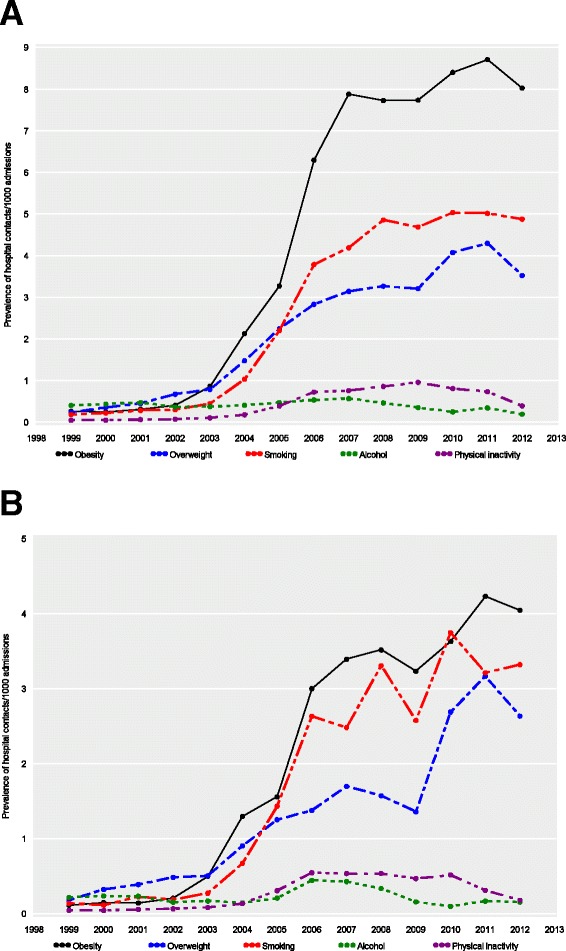
Annual prevalence of codes for overweight, physical inactivity, smoking or excessive alcohol consumption in the Danish National Registry of Patients per 1,000 hospital contacts overall (**a**) and for first-time contacts within 10 years, 1999–2012 (**b**)

Figure 1 shows the annual prevalence of lifestyle risk factors recorded in the DNRP per 1,000 hospital contacts overall (Fig. 1a) and for first-time contacts in 10 years (Fig. 1b). As appears, obesity was by far the most frequently diagnosed lifestyle risk factor with a marked 30-fold increase over time from 0.26 per 1,000 contacts in 1999 to 8.02 in 2012. This increase was particularly steep between 2003 and 2007, with an 8-fold increase from 0.86 per 1000 contacts in 2003 to 7.88 in 2007. Over the study period, we also noted a 26-fold increase in the diagnosis coding of smoking (from 0.18 to 4.88 per 1000 contacts) and a 14-fold increase in the coding of overweight (from 0.23 to 3.52 per 1000 contacts). Similar to obesity, the increase was steepest between 2003 and 2007. Thereafter, increases appeared to level off. In comparison, diagnosis codes for excessive alcohol consumption remained at a very low level around 0.40–0.50 per 1000 hospital contacts throughout the study period. Codes for physical inactivity were infrequently used but increased in the latter half of the study period from 0.39 per 1,000 contacts in 2005 to 0.73 in 2011.

The presence of lifestyle risk factors was less frequently recoded at first vs. subsequent hospital contacts (Fig. 1b). For first contacts, the prevalence of obesity increased by 36 fold (from 0.11 per 1000 in 1999 to 4.05 in 2012), overweight by 14 fold (from 0.18 to 2.63), and smoking by 23 fold (from 0.14–3.32). For first contacts, the increases did not level off after 2007.

Table 1 displays the prevalence of lifestyle risk factors recorded in the DNRP in 2012 according to type of hospital contact, geographical region, and patient age, sex and underlying disease. Most lifestyle factors were coded twice as often at inpatient than outpatient contacts. Overall, the prevalence of diagnosis coding varied by geographical region. In 2012 for instance, the prevalence of codes for obesity varied from 3.31 per 1000 contacts in the North Denmark Region to 21.37 per 1000 in Region Zealand. The prevalence of codes for obesity was markedly higher among females compared with males (10.76 per 1000 for obesity and 4.98 per 1,000 for overweight among females in 2012 vs. 4.22 and 1.50, respectively among males) whereas the prevalence of excessive alcohol intake was substantially higher among males (0.31 vs. 0.10 in females in 2012). Smoking codes were only slightly more prevalent among males (5.22 vs. 4.64 in 2012). For all lifestyle risk factors, the prevalence of recording was highest among adults below 50 years of age (Table 1).

**Table 1:** Annual prevalence of codes for overweight, physical inactivity, smoking, or excessive alcohol consumption in the Danish National Registry of Patients per 1,000 hospital contacts in 2012

	Obesity	Overweight	Smoking	Alcohol consumption	Physical inactivity
Overall	8.02	3.52	4.88	0.19	0.39
Type of contact					
Outpatient	5.19	2.39	3.28	0.12	0.33
Inpatient	16.71	7.00	9.75	0.40	0.58
Health care region					
Capital Region of Denmark	6.40	4.49	7.62	0.21	0.37
Region Zealand	21.37	4.93	4.64	0.14	0.04
Region of Southern Denmark	7.28	4.45	4.61	0.16	0.05
Central Denmark Region	4.83	1.23	1.88	0.25	0.78
North Denmark Region	3.31	1.85	4.97	0.11	0.83
Age group					
16–34	10.22	6.82	4.51	0.07	0.15
35–49	10.23	3.72	5.87	0.19	0.34
50–64	6.07	1.34	5.30	0.22	0.46
65–79	6.94	2.85	4.64	0.26	0.58
80+	6.91	3.99	2.84	0.19	0.30
Sex					
Female	10.76	4.98	4.64	0.10	0.36
Male	4.22	1.50	5.20	0.31	0.42
Underlying disease^a^					
Myocardial infarction	9.01	2.43	5.60	0.24	0.91
Congestive heart failure	11.24	3.15	5.55	0.45	0.41
Peripheral vascular disease	9.54	2.43	8.52	0.35	0.64
Cerebrovascular disease	7.81	2.80	5.81	0.40	0.34
Chronic obstructive pulmonary disease	12.95	5.74	14.11	0.44	0.46
Diabetes	15.64	3.20	5.90	0.30	0.46
Cancer	6.59	2.23	4.82	0.20	0.41
Liver disease	8.73	2.06	12.68	1.15	0.25

^a^The underlying disease is defined as the principal discharge diagnosis recorded in the Danish National Registry of Patients

In 2012, codes for obesity were prevalent among patients with diabetes (15.64 per 1000), COPD (12.95 per 1000), and congestive heart failure (11.24 per 1000). Codes for smoking were prevalent among patients with COPD (14.11 per 1000), liver disease (12.68 per 1000), and peripheral vascular disease (8.52 per 1000) (Table 1).

## Discussion

Our findings demonstrate that the nationwide recording of lifestyle risk factors in the DNRP is low but has increased substantially over the last 15 years, in particular for obesity, overweight, and smoking.

Our study design only allowed us to examine the recording of lifestyle in the DNRP. The optimal study would also examine sensitivity and specificity of the registrations, i.e., compared with a gold standard for presence or absence of each lifestyle factor in a given patient. Our estimates may reflect both changes in the actual prevalence of these lifestyle habits in the Danish population and the physician’s changing use of the available codes. Recent survey data showed that 47 % of the Danish general population are overweight and 14 % are obese, 17 % smoke daily, 16 % are physical inactive to a degree that may adversely affect their health, and 9 % drink more than the latest recommended maximum levels provided by The Danish Health and Medicines Authority [12]. Thus, in comparison our estimates suggest that the completeness of registration of lifestyle habits in Danish hospitals is very low, not least because patients with hospital contact with acute and chronic diseases generally have a higher prevalence of unhealthy lifestyle factors than the general population [13–15]. This low completeness is especially troubling given the increasing prevalence of obesity, sedentary lifestyle, and lifestyle related chronic noncommunicable diseases nationally [16, 17]. For example, primary data show that 88 % of patients with newly diagnosed type 2 diabetes in Denmark are either overweight or obese [18], compared with 2 % of patients with diabetes coded with overweight per DNRP contact in our study. Among patients with COPD seen in Danish outpatient clinics, 33 % are known to be active smokers and 64 % are former smokers [19], compared with a prevalence of recorded tobacco smoking in the DNRP in 2012 of only 14.11 per 1000 contacts among patients with COPD in our study.

Systematic patient assessment and documentation is important to ensure that risk factors are identified and that all patients are offered relevant counselling and intervention when needed. The documentation is vital to inform subsequent investigations, treatment, care and follow up [20]. The recording of lifestyle risk factors is a simple means to ensure communication across the health care system (e.g., when discharging a patient from hospital with referral to primary care). Failure to record and communicate information about a patient’s adverse lifestyle at hospital level could in this context be seen as a missed opportunity for ensuring continuity in care in general and in risk factor management in particular. In worst case, primary care physicians may perceive this lack of attention to life style factors as an indication that the hospital does not endorse risk-lowering interventions. This could potentially have serious implications since the primary care sector, due to the high population reach [21] and the patients’ general acceptance of the role of primary care providers in preventive care [22], play a key role in implementing life style modifying interventions. The Danish National Board of Health has aimed at strengthening the prevention of lifestyle-related diseases through systematic registration of overweight, smoking, excessive alcohol use, and physical inactivity in hospital medical records of hospitalized patients [11]. Subsequently, the screening was also included in the Danish accreditation standards, which have been mandatory at all public hospitals since 2009 [9]. The increasing use of codes for obesity, overweight, and smoking since 2003 may reflect these initiatives, although increases in the underlying prevalence of e.g. overweight among patients with hospital contacts may also have contributed. However, despite the formal requirements and the existing knowledge on the importance of lifestyle risk factors for treatment and prognosis, systematic recording of lifestyle in the hospital setting remain underutilized. A range of factors may explain this phenomenon. First, the physician and hospital department treating the patient may be reluctant to prioritize the recording due to time constraints and existing demands for recording of a widespread range of other data in relation to each patient contact. Second, the low priority given to recording of lifestyle risk factors may be supported by the fact that individual physician or hospital department will not experience any immediate benefit from their efforts. Third, there are no financial incentives to record the information. A more complete recording in the DNRP will require that these challenges are addressed, e.g., by ensuring more simple and user-friendly IT systems and a revision of the hospital reimbursement system to take into account individual patient characteristics such as adverse lifestyle that may prolong hospital care, complicate treatment, and ultimately influence patient outcomes.

We were unable to assess whether physicians, nurses, or other caregivers actually had asked for lifestyle risk factors and offered counseling to their patients, without coding the presence of any lifestyle risk factor in the DNRP. Since the early 2000s, the Danish health care authorities have initiated continuous monitoring of the quality of care provided by all Danish public hospitals to patients with a number of important diseases, including diabetes, COPD, heart failure, stroke, and cancer [23]. Nationwide clinical quality of care databases now exist for more than 60 different diseases and conditions in Denmark [24]. At least 10–15 of these quality databases systematically collect primary data on body mass index, smoking, and alcohol intake for most (>80 %) of their patients. The databases include patients with any hospital contact with e.g. diabetes in the Danish Diabetes Database for Adults [25], stroke in the Danish Stroke Registry [26], and outpatients with COPD in the Danish Clinical Register of COPD [19]. For a few of these conditions (e.g. COPD outpatients, or patients with hip fracture), selected lifestyle data are actually recorded directly via codes in the DNRP. This fact likely explains the increasing prevalence of e.g. diagnosis codes for tobacco smoking observed in patients with COPD in our study. For other conditions, the nationwide set-up with good coverage of several lifestyle risk factors for the patients in dedicated clinical databases illustrate the discrepancy between the assessment of lifestyle risk factors in everyday clinical practice, and the recording of this information in the DNRP.

## Conclusion

In conclusion, our study shows that – compared with knowledge from population surveys and clinical quality databases – the completeness of diagnosis codes for lifestyle risk factors recorded in the DNRP is very low. Since unhealthy and modifiable lifestyle risk factors are strong determinants for the incidence and development of leading causes of morbidity and mortality, more complete data on these factors should be a national health care priority as it may assist in targeting preventive efforts.

## Additional file

Additional file 1:
**International Classification of Diseases (ICD) and treatment codes used in the study.** (DOC 35 kb)
