# Supplementary material for: Evidence for the low recording of weight status and lifestyle risk factors in the Danish National Registry of Patients, 1999–2012
Source: BMC Public Health. 2015 Dec 30;15:1320. doi: 10.1186/s12889-015-2670-9 (PMC4696325; doi:10.1186/s12889-015-2670-9)
Supplement: Additional file 1: — International Classification of Diseases (ICD) and treatment codes used in the study. (DOC 35 kb) [file 12889_2015_2670_MOESM1_ESM.doc]

International Classification of Diseases (ICD) and treatment codes used in the study

| **Lifestyle codes:** |  |
| --- | --- |
| Obesity | ICD-10: E660B-H, E67.8. E68 |
|  | Treatment codes: BQFT03, BQFS03 |
|  | Administrative procedure codes: ZZ0242 + (VPH0030-VPH0050, VPK0030-VPK0050) |
| Overweight | ICD-10: E660A |
|  | Administrative procedure codes: ZZ0242 + (VPH0025-VPH0029, VPK0025-VPK0029) |
| Smoking | ICD-10: F17, Z720A, Z720E |
|  | Treatment codes: BQFT01, BQFS01 |
|  | Administrative procedure codes: ZZP01A1A, ZZP0020 |
| Alcohol consumption | ICD-10: Z721 |
|  | Treatment codes: BQFT02, BQFS02 |
| Physical inactivity | ICD-10: Z723 |
|  | Treatment codes: BQFT04, BQFS04 |
| **Primary discharge diagnoses:** | |
| Myocardial infarction | ICD-10: I21, I22, I23 |
| Congestive heart failure | ICD-10: I50, I11.0, I13.0, I13.2 |
| Peripheral vascular disease | ICD-10: I70, I71, I72, I73, I74, I77 |
| Cerebrovascular disease | ICD-10: I60-I69, G45, G46 |
| Chronic obstructive pulmonary disease | ICD-10: J41 -J44 |
| Liver disease | ICD-10: B15.0, B16.0, B16.2, B18,B19.0, K70.0-K70.4, K70.9, K71, K72, K73, K74, K76.0, K76.6, I85 |
| Diabetes | ICD-10: E10.0, E10.2-10.9, E11.0-E11.9 |
| Cancer | ICD-10: C00-C96 |
